# A metabolic signature of colon cancer initiating cells

**DOI:** 10.1186/2049-3002-2-S1-P32

**Published:** 2014-05-28

**Authors:** Christelle Johnson, Kai-Yuan Chen, Xiaojing Liu, Pengcheng Bu, Jason Locasale, Xiling Shen

**Affiliations:** 1 School of Electrical and Computer Engineering, Cornell University, Ithaca, NY, USA; 2 Division of Nutritional Sciences, Cornell University, Ithaca, NY, USA; 3 Biomedical Engineering Department, Cornell University, Ithaca, NY, USA

## Background

Colon cancer initiating cells (CCICs) play important roles in colorectal cancer (CRC) tumorigenesis. CCICs exhibit certain stem cell-like features, including self-renewal, differentiation, and asymmetric division. CCICs are often identified by their expression of marker CD133 among other markers. However, it has remained largely unclear whether CCICs isolated from different CRC tumors share common mechanisms that account for their phenotype, or are completely different cells that were categorized simply by their tumorigenic capacity.

## Materials and methods

We set out to address this question by first analyzing five microarray datasets accessed through the NCBI Gene Expression Omnibus (GEO) that measured the transcriptome of CD133+ versus CD133- CRC cells. Using patient-derived CRC lines previously established in our lab, we performed unbiased metabolomics analysis to identify the metabolic signature of CD133+ CCICs by high-resolution mass spectrometry. Bioinformatics, metabolic pathway enrichment analysis, and KEGG pathway online module were then applied to integrate transcriptomic and metabolomic data.

## Results

The transcriptome analysis suggested that CD133+ cells consistently regulate certain genes differentially from CD133- cells (3178 genes, p <0.05). Pathway analysis on the curated gene set highlighted the enrichment of metabolic pathways, pathways in cancer, and transcriptional misregulation in cancer. Metabolomics studies on CD133+ and CD133- cells showed differential metabolite levels between the two groups (54 metabolites, p <0.05). The analysis identified carbohydrate metabolism (glycolysis, TCA cycle) and cysteine and methionine metabolism as consistently altered in CCICs, with enzyme expression matching the corresponding metabolite levels.

## Conclusion

Our system-level transcriptomic and metabolomic analyses on various colorectal cancer sources unraveled a distinct metabolic signature of CD133+ CCICs that involve glycolysis, the TCA cycle, and cysteine/methionine metabolism. The identified metabolic signature provides insights into reported stem cell-like properties of CD133+ CCICs. The involved metabolic enzymes and metabolites may potentially serve as biomarkers for disease diagnosis and prognosis, and therapeutic targets for CRC treatment.

